# Trends in the travelers’ demand for pre-travel medical advice at a Spanish International Vaccination Center between 2000 and 2017

**DOI:** 10.1371/journal.pone.0217588

**Published:** 2019-05-30

**Authors:** Marina Segura, Rosa Lopez-Gigosos, Eloisa Mariscal-Lopez, Mario Gutierrez-Bedmar, Alberto Mariscal

**Affiliations:** 1 International Vaccination Center, Medical Service, Port Health Authority of Malaga, Malaga, Spain; 2 Department of Public Health and Psychiatry, Faculty of Medicine, University of Malaga, Malaga, Spain; Yenepoya Medical College, Yenepoya University, INDIA

## Abstract

Crises and disasters affect the numbers of people traveling either for tourism or other reasons. Many studies have been published on the effects of such events on travel, especially on tourism, and based on the arrivals or departures of travelers to or from countries. Our aim was to assess the influence of these events on the demand for pre-travel medical consultation in an International Vaccination Centre (IVC). Data on 94683 international travelers who visited 113529 international destinations attended at the IVC of Malaga (Spain) during 2000–2017 were studied. A descriptive and time series analyses was conducted. The demand to IVC was 3.47 times higher in 2017 than in 2000. The increase has not been the same for all destinations: Travel to South-East Asia and Western Pacific World Health Organization (WHO) regions has multiplied by 10, while in the same period, Africa WHO region has declined from 36% to 20% of total demand. Thailand, India and Brazil were the countries with the highest demand (21% of all pre-travel consultations). We found out three periods, concurrent with some socioeconomic or health events, in which the number of travellers attend decline with respect to the previous years, or the growth was very slow. Growth in the demand for pre-travel medical advice in parallel with a foreseeable increase in the number of travelers is expected. Pre-travel medical services must be adapted to this increase. This study of the trend of demand for pre-travel medical information should new related problems to travel to be identified and quantified, and should assist improvement of policies and programs aimed at care of travelers.

## Introduction

During the past six decades, international travel, especially tourism, has undergone unprecedented growth and diversification. Economically, tourism has become one of the most important and fastest growing sectors in the world.

The World Tourism Organization (UNWTO) forecasts that international tourist arrivals worldwide will grow 3.3% annually between 2010 and 2030, reaching 1.8 billion visitors to foreign countries in 2030[1]. It is further expected that such arrivals at emerging and developing countries (up 4.4% annually) will double those at advanced economies (up 2.2% annually). The market share of emerging economies is expected to reach 57% by 2030 (compared with 30% in 1980 and 45% in 2016)[1,2]. Knowledge of the evolution and trends of international travel provides valuable information for developing strategies and actions aimed at addressing, for instance, issues related to travelers’ health, including accidents, food safety, and primarily infectious diseases, such as those transmitted by insects. It must also be ensured that tourism growth is comprehensive and sustainable, and that the receiving communities and arriving travelers are protected[3].

During travel, travelers are often exposed to unfamiliar physical and environmental conditions or unknown risk factors. Travel medicine professionals must respond to travelers accurately and individually concerning the health risks associated with travel, and provide preventive measures that include information on recommended vaccines, malaria chemoprophylaxis, health education on water and food, measures to protect against insects or related to disease transmission through sexual contact, air or animal bites, or even self-treatment measures for diarrhea[4].

For those traveling to a country considered to pose high levels of health risk, travel medicine providers should perform a careful assessment of each traveler’s itinerary, including destinations, duration and season of travel, and potential activities, as well as the traveler’s personal characteristics (e.g., age, sex, educational level, medical history, previous vaccines). Other factors such as travelers’ education and culture, regions’ economic conditions, political problems, and tourism offers, as well as environmental and humanitarian crises, will also have a strong influence on proper continued development of travel. However, although most countries have centers specializing in health information for international travelers, it is estimated that only 10%–50% of travelers seek to obtain information and/or vaccines at these center to reduce travel-related health risks[4,5].

In Spain, preventive care for international travelers (health education and vaccination) is mostly conducted through a network of 107 public International Vaccination Centres (IVCs) and about 10 private centers. Throughout the period of the present study (2000–2017), the annual number of travelers that required attention at the IVCs grew, reaching 300,000 in 2017[6].

Despite this high number of travelers, few population-based data exist detailing the travel characteristics, pre-travel health preparation and health-related behaviors of international travelers who visit the IVCs. In a study of global tourism, the researchers found lower numbers of international trips coincided with two events that had widespread global impact: the severe acute respiratory syndrome (SARS) epidemic in 2003 and the international financial crisis of 2009[2]. The IVC of Malaga (IVCMa), which serves a large number of international travelers, piloted an analysis of time series data to study behavior over time in the demand for pre-travel health care and the influence of events of international impact on that demand.

Improving health care provision for international travelers to prevent travel-related diseases, and adapting international vaccination services to meet demand, requires continuous analysis of situations. Adequate planning of these resources is impossible without exhaustive knowledge of trends in travel. Detailed analysis of the trends and characteristics of travelers required attention at the IVCs could be useful for adapting resources to meet the growing needs surrounding international travel.

The aim was to use the data collected from the people who have requested attention in the IVCMa to know aspects as annual and seasonal shifts in demand for information pre-travel, trends in destinations, and its possible relationship with some international events (e.g., economic and political crises, global epidemics, humanitarian catastrophes). This baseline population-data on travelers can enable estimation of the repercussions of international adverse events on trends in destinations and traveler types, which in turn facilitates ongoing adjustments of the IVCs to meet travelers’ demands.

## Methods

The IVCMa, in southern Spain, covers a population of approximately one million residents, and is the only information center for travelers in the region. In the past 3 years, 9,000–10,000 travelers a year have visited it, accounting for approximately 3%–5% of people accessing the 107 IVCs nationwide.

Travelers requesting pre-travel medical care in the IVCs receive mandatory and/or recommended vaccines, and advice related to water and food measures, prevention of mosquito bites, animal bites and other adapted recommendations to the profiles of the trips and the travelers. According to the destination and the specific characteristics of the trip, the vaccines prescribed at the IVCMa are mainly: yellow fever, meningitis, typhoid fever, cholera, hepatitis A and B, Japanese encephalitis, influenza, tetanus-diphtheria, whooping cough, rabies, poliomyelitis, measles, mumps, rubella and tick-borne encephalitis.

The Foreign Health Information System (SISAEX) is an administrative database management system implemented in 2000 that collects data on travelers requesting travel-related health information at all IVCs. The variables collected by the SISAEX at the individual level (for each traveler) are: age, sex, destination, date and duration of the trip, type of trip, clinical data (diseases, allergies, treatments), previous vaccines, vaccines administered in the IVC, recommended vaccines and antimalarial prophylaxis.

For the study, only the aggregated monthly data are extracted: the monthly and annual number of travelers attended, the monthly and annual number of countries visited.

The monthly travel destinations were introduced as a new variable in the SISAEX database in 2005. The Ministry of Health, Consumption and Social Welfare of Spain manages and administers the SISAEX system.

Although most inquiries at IVCs are related to travel to destinations that have health risks, there are also inquiries from travelers to countries not considered at risk. These commonly are travelers who are required or recommended to be vaccinated against meningitis or tuberculosis because they will be attending educational institutions or working in hospitals or clinics. Our study considered a destination health risk assignment when one of the countries visited was classified by WHO as having high or medium risk of hepatitis A virus infection[7]. Travelers typically go to IVCs on the recommendation of travel agencies, advertisements or media campaigns related to the preparation for trips to health risk countries, or on the recommendation of friends, travel websites or other information sources.

The monthly number of travelers personally attended to at the IVCMa, according to the SISAEX database, was used in a descriptive study with ecologic time series design and carried out between January 2000 and December 2017.

The data were obtained from medical records in a fully anonymized and de-identified manner. The information obtained from the SISAEX and used for this study is not personal, only monthly data series (number or proportions) of travelers that do not allow the identification of any user. The authors had not access to identifying information at any moment. In addition, all international travelers who are attended at the IVCs, previously request a pre-travel medical advice by phone or web are informed that their data will be used to centralize sanitary control procedures and can be used for statistical purposes and health research.

Initially, a graphical exploration was planned for the purpose of globally visualizing the activity of the monthly data series. This was considering a time series is defined as a succession of observations corresponding to a variable at different times and observed at regular intervals of constant duration. To control the level of quality, two of the authors edited the master databases to standardize the data and ensure reliability of subsequent analyses. After analyzing aspects such as trends and seasonality of the number of travelers over the study period, a modeling process was developed using an autoregressive integrated moving average (ARIMA) model. This was to expand on a simplified representation of the most important characteristics of the data series.

The number of travelers visiting the IVCMa (hereinafter “TAs”) increased almost continuously over the study period, although some stages of deceleration or negative growth were seen. The relationship between the TA number and high-impact international events that occurred between 2000 and 2017 was taken into account.

Unforeseen impacts from internationally impactful events, such as terrorist attacks, political instability, health pandemics, natural disasters, or humanitarian and socioeconomic crises, were considered. The primary sources for tracking and detection of such events included academic studies, articles, reports, press releases, books, journals and data from relevant websites; all generally related to the tourism industry. The Emergency Events Database (EM-DAT) was especially useful in our search. EM-DAT was launched in 1988 by the Centre for Research on the Epidemiology of Disasters, with the initial support of the WHO and the Government of Belgium, to facilitate humanitarian action at the national and international levels.

Analysis of descriptive statistics was performed with Microsoft Excel 2011 and IBM SPSS 22.0.

## Results

A total of 94,683 people requested pre-travel medical advice at the IVCMa between January 2000 and December 2017. Table 1 shows the annual distribution of the number of TAs, the number of countries about which they requested information, along with the number of medical professionals attending the pre-travel consultations, and the number of vaccines prescribed for each year studied. From the beginning of the study until 2017, the number of TAs increased by 325.2%, and vaccines by 576.8%. However, between 2000 and 2004, SISAEX only gave data on the monthly number of travelers and prescribed vaccinations, though not on destinations; therefore, in Table 1, the number of travelers and destinations is the same for that period. From 2005, monthly number of travel destinations were included in SISAEX, although between 2005 and 2007, some faults in the recording system led to losses ranging from 3% in 2007 to 10% in 2005 and 2006 for destination data. In the following years, there were no such losses and all destination countries were recorded.

**Table 1:** Travelers attended to and prescribed vaccines at the International Vaccination Centre of Malaga.

Year	Travelers (N = 94683)	Destinations^1^ (N = 113529)	Number of doctors in IVCMa	Prescribed vaccines (N = 267766)	Prescribed vaccines per traveler (mean)
2000	2885	2885^2^	1	5253	1.82
2001	2852	2852^2^	1	6111	2.14
2002	2549	2549^2^	1	5294	2.08
2003	2306	2306^2^	1	4713	2.04
2004	2826	2826^2^	2	3790	1.34
2005	2956	2499	2	7410	2.51
2006	3772	3405	3	8970	2.38
2007	4866	4874	3	14384	2.96
2008	4593	5543	3	13183	2.87
2009	5262	6763	3	15779	3.00
2010	5173	6872	4	16289	3.15
2011	5442	7085	4	17485	3.21
2012	6516	8041	4	20562	3.16
2013	7701	8763	4	23922	3.11
2014	7656	8646	4	22189	2.90
2015	8038	9304	4	23236	2.89
2016	9273	13133	4	28896	3.12
2017	10017	15183	4	30300	3.02

^1^ Number of countries for which information was requested

^2^ Between 2000 and 2004. SISAEX did not collect information on the monthly number of countries visited.

**Fig 1:**
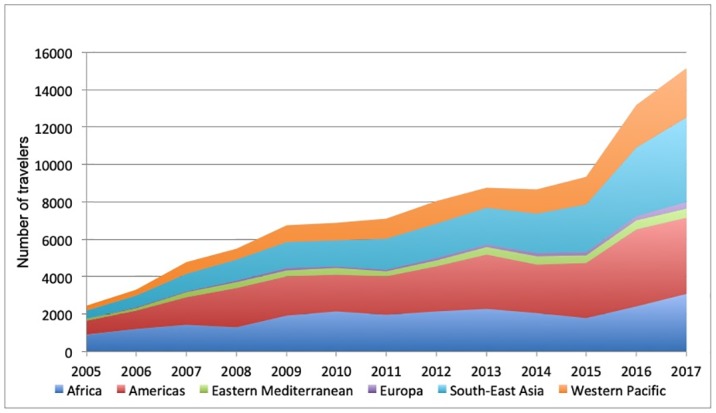
Destinations of travelers by WHO region and by year.

A total of 94,683 TAs requested health information on at least 113,529 countries between 2000 and 2017 (a mean of 1.2 countries per traveler). The WHO Americas Region was the most common destination (29.9%) followed by the WHO African Region (24.2%), WHO South-East Asia Region (23.6%), and WHO Western Pacific Region (14.5%). Fig 1 shows the annual distribution of TA destinations since 2005. Although the demand for pre-travel information grew with respect to all destinations, a greater increase was observed for the South-East Asia and Western Pacific regions. In 2017, the total demand for information for these two regions was 47.3% (28.8% in 2005). However, in the WHO African Region, demand in relation to total annual demand declined from 36.8% (2005) to 20.4% (2017).

**Fig 2:**
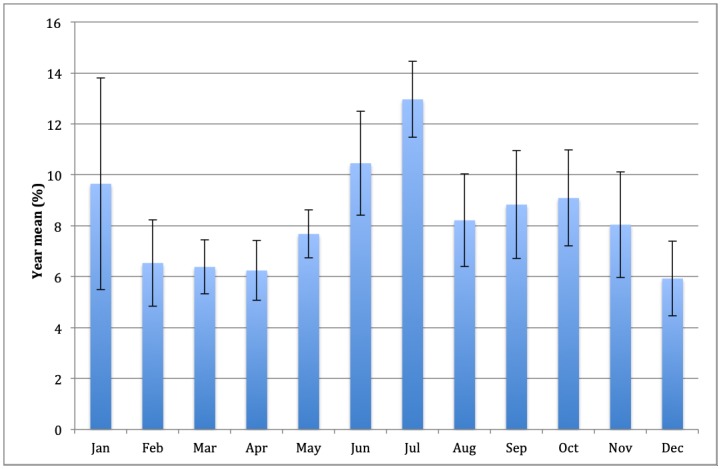
Monthly distribution of travelers, 2000–2017. Each bar represents the average annual percentage for each month and the Y-error bars are the corresponding standard deviation.

Seasonal variation was observed when analyzing the number of TAs per month during the study period. The months of June and July had the highest numbers of traveler consultations (Fig 2). The monthly distribution of TAs was similar throughout the study and showed no significant differences across the years.

**Fig 3:**
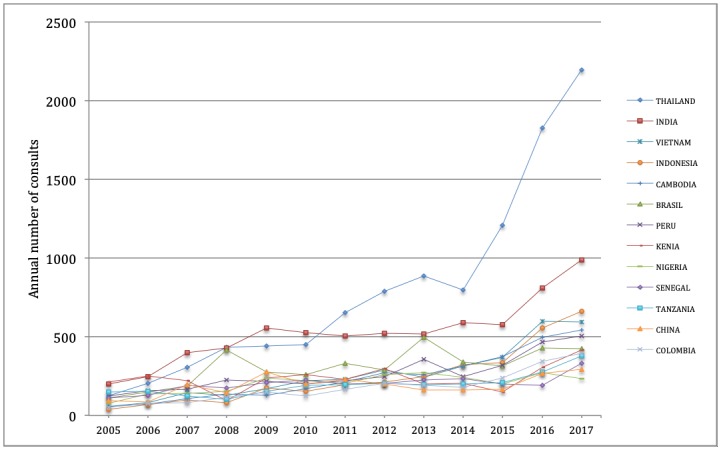
Destination countries with highest demand for pre-travel health information between 2005 and 2017 at the International Vaccination Centre of Malaga (51.8% of consultations).

Between 2005 and 2017, TAs requested pre-travel health information on 120 different countries, although 50% of the consultations were about just 13 countries (Fig 3). Most pre-travel consultations (97%) concerned countries categorized as posing a health risk, while the remainder (3%) regarded countries deemed to pose no health risk (Australia, Canada, European countries including Russia, Japan and New Zealand). Thailand, India and Brazil were the three countries on which the most pre-travel information was requested, with 21,229 queries (21% of all consultations) in the 13 years recorded. Over this period, only India and Thailand remained among the four most visited countries during all the years. However, in the final 3 years, demand for pre-travel information to WHO Southeast Asia Region countries underwent a conspicuous increase; Thailand, India, Vietnam, Indonesia and Cambodia accounted for more than 32% of TA destinations between 2015 and 2017.

**Fig 4:**
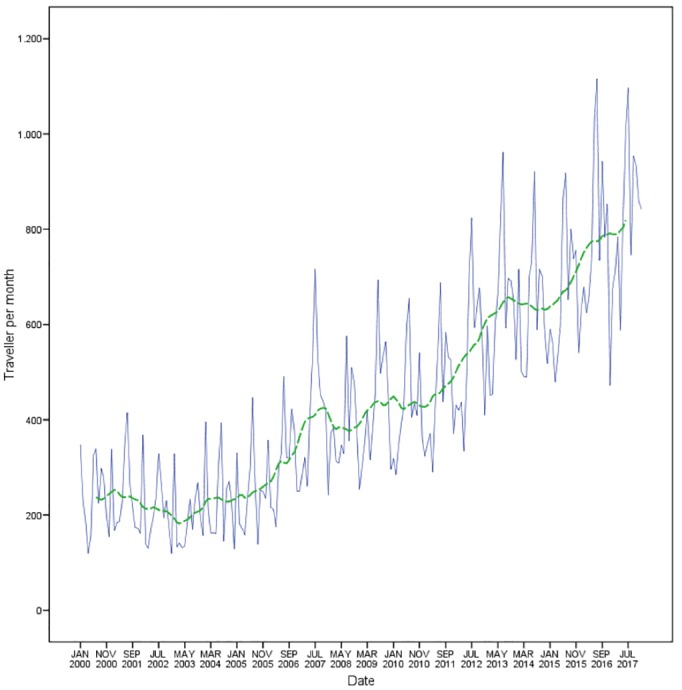
Travelers attended at the International Vaccination Centre of Malaga. Seasonally adjusted travelers (12th order moving average model denoted by ARIMA model) (green) and original data (blue), 2000–2017.

As Fig 4 shows, the number of TAs increased approximately fourfold since 2000, with strong seasonality within each year. This increase was continuous, except for during three primary slow periods: in 2001, 2007 and 2013. Throughout the years studied, most of the countries had regional or global challenges or crises of diverse nature and severity, which could account for the trend observed in the number of TAs. Table 2 shows a timeline of some of the main events affecting international travel between 2000 and 2017.

**Table 2:** Timeline of some of the most well-known events that could affect international travel between 2000 and 2017.

NY Terrorist attacks	2001
Bombing Bali.	2002
SARS	2003
Madrid Terrorist attacks, Earthquake & Tsunami Indian Ocean, Avian Flu	2004
Bombing Bali	2005
International Financial crisis.	2007–2008
Flu pandemic	2009
Earthquake Haiti	2010
Spanish unemployment rate >20%	2010–2015
Arab Spring	2010–2012
Ukrainian crisis	2013
Ebola outbreak. Daesh	2014
Europe refugee crisis	2015

A temporary association between lower TA numbers and terrorist attacks, health or financial crises, and environmental catastrophes can be observed. The chronology of other political, social, terrorist, climatic or natural disaster events that could also have been included in the number of traveler requests was taken into consideration and is discussed later.

## Discussion

This is the first population-based study that used the SISAEX database to analyze specific variables related to demand for health information at an IVC in Spain. For the period 2000–2017, the demand for pre-travel consultation at the IVCMa increased 347%, from 2,885 travelers (2000) to around 10,000 in 2017 (Table 1). The IVCMa services one of the largest internationally traveling populations in Spain; it accounted for approximately 3%–5% of all annual international pre-travel consultations by residents in Spain. Between 2000 and 2017, the number of trips abroad increased 420% across all of Spain’s population[8]. This increase, however, was not continuous throughout the study period, with years in which the number of travelers decreased year-on-year and others with increases of more than 30% (2005–2006 and 2006–2007). Between 2015 and 2017 the average increase in TAs at the IVCMa was 10%; an increase higher than the 3.9% recorded worldwide in the number of international tourist arrivals (overnight visitors) in the same period[1]. However, travelers who requested information at the IVCs are almost exclusively those who will travel to countries that pose health risks. Thus, when taking the UNWTO region visited into account, the increase at the IVCMa was similar to that for UNWTO Asia and the Pacific (9%) and Africa (8%) regions[1].

Despite this, the average number of prescribed vaccines per traveler gradually increased from 1.8 to 3.0 over the course of the study. This increase resulted from introduction of new vaccines of interest for travelers to at-risk countries, such as the serogroup C meningococcal vaccine (2000) or more recently the serogroups ACWY meningococcal vaccine (2010) and serogroup B vaccine (2015)[9]. Additionally, there were no specific vaccines against the SARS coronavirus (CoV) that emerged in southern China at the end of 2002, or 10 years later MERS-CoV in the Middle East, or human influenza A (H5N1) in 2006. However, travelers were recommended (especially in 2006) to receive the trivalent human influenza vaccine to avoid confusion of the usual flu symptoms with others flu, or in the case of avian influenza, to prevent a potential mix of human and avian strains of the influenza virus[10].

The greatest numbers of trips in European countries take place during the summer in the most common holiday period, which is between the months of July and August [2,11,12]; therefore, increased demand is expected for pre-travel health information in the preceding months (June and July). The strongest demand for pre-travel information at the IVCMa also occurred between June and July (Fig 2), with an average of 23.4% and standard deviation (SD) of 3.1 of annual demand for the period 2000–2017. This seasonality in demand for health information is similar to tourism demand of European Union residents who, in August and July 2015 and 2016, made approximately 23% and 26%, respectively, of all their trips for the year[11,13]. Between 2000 and 2011, 33.3% (SD 0.93) of trips by EU residents were taken in the third quarter of the year[14]. However, according to data from the Spanish National Institute of Statistics (INE) and Eurostat[15], in some years a reduction in trip seasonality is observed; therefore, the differences between the months of greatest and least demand are proportionally smaller. In this manner, in 2016, an increase of 4% in the number of Spanish residents traveling internationally was observed, and 35% (5,503,892) travelled between July and September, which was down 3.4% from the same period in 2015[8]. In the present study, this reduction in seasonality was not observed. The average annual percentage of travelers served between June and July was 23.4% (SD 3.1) and when comparing annual means, a statistically significant difference was not observed. This could be explained by the fact most trips to areas for which a previous visit to an IVC was recommended, are made between July and September. These months are more conducive to travel, as they coincide with school and other institutional holidays, leading to more pursuit of tourism in Spain and in most of the countries of the northern hemisphere[16]. These trips tend to be also longer than those that do not require prior medical information and can be taken at any time of the year. Weather conditions and forecasts might also influence the holiday length. Moreover, in some destinations, July or August are the best times to avoid adverse weather such as intense rains (e.g., monsoons) or risk of hurricanes and tropical storms[17,18].

In the years analyzed in our study, three periods can be observed in which the number of TAs did not increase year-on-year, or the growth was slight (Fig 4). Tourism is generally affected by local problems of varying etiology and is difficult to predict, or is affected by the current economic conditions of developed and emerging destination countries[19]. Tourism demand is highly sensitive to terrorist attacks and political violence, as tourists—similar to most anyone—value tranquility and peace to enjoy the pleasures and activities that destinations offer[20]. The September 11, 2001, terrorist attacks on New York City and Washington, D.C., as well as a series of other terrorist attacks between 2001 and 2005, led to a decrease in tourism worldwide[21]. In our study, the number of travelers recorded in 2000 was not again reached until 2005, and new declines were observed in 2008 and 2014. However, other, non-political crises occurred both in the first interval and later. Health-related crises, such as those in 2003 and 2009, respectively due to SARS and influenza A (H1N1), and more recently the Ebola outbreak in 2014, attracted greater worldwide media attention. This was because they were new diseases, easily transmitted, had high capacity to spread and high mortality, and also had a negative effect on international tourism[22–26].

Natural disasters, with unpredictable and catastrophic impacts, such as the 2004 Indian Ocean earthquake and tsunami, volcanoes, and flooding caused by hurricanes and typhoons, also impact tourism. Although their effect can be limited over time, there can be widespread cancellation of trips, not because of fear of recurrence, but rather because of the catastrophe’s impact on the destinations’ infrastructure[27].

In recent years, the global economic and financial crisis of 2008–2009 was the international event that has most affected tourism, triggering negative growth in all types of tourism[12]. In Spain’s population, the economic crisis not only had a negative impact on the departure of tourists, but also produced a change in the types of travel and traveler, and increased work-related travel during the years of the crisis and later on[28].

Throughout the present study and coinciding with the periods in which there was a decline in the number of TAs, different crises clearly occurred at small intervals of time and almost simultaneously. It is extremely complicated, if not impossible, to determine the responsibility of one or more such crisis for the decrease of travelers at a global level. It is expected that when an event that impacts tourism is highly local in nature, such as an earthquake, cyclone or political conflict, tourism to that area will decrease, but globally, travelers will only change their destination; thus, the final balance of travelers will not be affected.

The countries involved in the Arab Spring movement also experienced a sharp decrease in inbound tourists. This was attributed to the escalation of tension and continued insecurity in the region, especially between 2010 and 2011[29]. Now, conflicts in North Africa since mid-2015 have shifted demand to countries in southern Europe, Asia and the Caribbean, which give similar leisure offers[30]. Local crises have an impact on tourism to areas where they occurred, or to nearby areas, such as with the Ebola outbreak in the Gambia[26], terrorist attacks[31] and oil spills[32].

Throughout the period of the study, a large increase in requests for pre-travel health information related to Southeast Asian destinations was observed, especially in recent years. Between 2005 and 2017, demand at the IVCMa for advance information related to the WHO South-East Asia and Western Pacific Regions grew tenfold, from a total of 712 queries in 2005 (28.8%) to 7,165 in 2017 (47.3%). In fact, in 2017, Thailand was the country for which the largest numbers of requests health information were received (14.5% of all annual consultations). Growth of Asian destinations is highly conspicuous in UNWTO projections for 2030[33]. In the EU, trips to Thailand account for 3% of all destinations outside the European Community. In recent years, the Asian region is leading growth of international tourist arrivals; up 9% through September 2017, according to UNWTO figures[15]. The countries of the Mekong sub-region saw inbound tourist arrivals increase from 31.1 million in 2010 to 51.9 million in 2015 (Myanmar was up 490%, Cambodia 91.5%, Thailand 87%, Laos 72% and Vietnam 52%). Such growth for Asian destinations reflects travelers’ perceptions of safety, and the possibility of making cheaper, non-organized trips; therefore, this growth is expected to continue in the coming years[15,34–36].

Because the items studied were based on responses from travelers obtained before their trip, there is a chance of incorrect reporting, modifications of plans, or even cancellation, which may have introduced bias into the results. However, for the SISAEX data, personal responses were given directly to questions from the attending doctor at the IVC, after previously making an appointment. Respondents were then personally informed about recommended prophylaxis depending on their destination. Finally, depending on this consultation, they could receive a necessary vaccine and pay a predetermined fee. Therefore, we expected the presence of any misleading data was limited. In addition, if such data did emerge, the amount would be considerably small in relation to the scale of the total sample studied. Another possible limitation was that we had no data on those who did not go to the IVC to receive health information for their trip, or they received information via other means, such as friends, websites, books or reviews. Finally, our results reflect the internationally traveling population of Malaga between 2000 and 2017 and may not be representative of the traveling population of Spain.

As indicated above, between 2000 and 2004, the only available data were on the number of travelers and prescribed vaccines, and not on the destinations. Additionally, between 2005 and 2007, faults in the recording system resulted in some loss of data on travelers’ destinations. Thus, the analysis of trends in destination countries was limited to the period for which data were available (2005–2017). The lost destination data were also not substantial enough to have considerably affected the full data in this study.

Establishing a causal relationship between a reduced number of travelers and certain international crises is a complicated task. Most trips to at-risk areas are planned 6 months or longer in advance. Thus, although a crisis can attract a high degree of media attention at a certain time, after several months, travelers’ perceptions may have changed considerably. In this way, when terrorist events are not repeated, the tourism industry can recover in a period of 6–12 months[37]. Despite this, because different crises can occur simultaneously in a given country or region, even if using some previously described methods to endogenously determine the effect of individual crises on precise dates[26], it is impossible to know whether a single cause, or several at the same time, were responsible for a tangible effect on international tourism demand.

This study on the demand for pre-travel health information at the IVCMa provides valuable surveillance data on international travelers residing in southern Spain. Apart from that, the data on the frequency of travel and on destinations as extracted from SISAEX data elucidate could improve understanding of the relationship of the trend in the demand for pre-travel health information and crisis and events that impact tourism. These data together with other data from SISAEX that will soon be incorporated into the system (travel motive, demographic, socioeconomic, health variables) not analyzed in this study will enable determination of socioeconomic indicators on international travelers to health risk areas, and their needs in relation to pre-travel preventive measures. The data collected over a long period and analyzed in relation to crises and events that impact international travel will allow us to examine trends over time and take measures to anticipate travelers’ demands for pre-travel care. Additionally, a sharp increase in the number of travelers, and consequently in the demand for pre-travel health advice, can be expected in accordance with forecasts of international organizations concerned with travel and tourism. This will impel reinforcement of teams of medical professionals at ICVs, especially in periods of high demand. It may also lead to creation of alternative sources of education and vaccination for travelers, which could give potential solutions to anticipated overloads of traveler demand. Different levels of risk should be identified in accordance with the destination, type of travel and traveler; these should be initially identified to adapt the type of care and increase efficiency among resources. Based on the results of this research, it is uncertain to predict the impact of crises or other events on the number of travelers requesting pre-travel medical information, hindering progress towards a general model that allows adapting the resources of medical services from the trip to the demand. Additional studies on the evolution of the demand for pre-travel care, and incorporation of new additional questions, as well as conducting of post-travel studies through surveys given to travelers who previously visited IVCs, should allow us to identify and quantify new travel-related problems and help to improve policies and programs for providing care for travelers.

## Supporting information

S1 Tables Annual distribution of travelers by WHO region. (XLSX)

S2 Tables Monthly distribution of travelers as average annual percentage. (XLSX)

S3 Tables Annual number of travelers to the most visited countries. (XLSX)
